# Research and publication trends on knee osteoarthritis and cellular senescence: a bibliometric analysis

**DOI:** 10.3389/fphys.2023.1269338

**Published:** 2023-11-17

**Authors:** Shuai Wang, Jiyong Yang, Ruian Xiang, Congcong Li, Junyi Li, Xingxing Shen, Wengang Liu, Xuemeng Xu

**Affiliations:** ^1^ The Fifth Clinical College of Guangzhou University of Chinese Medicine, Guangzhou University of Chinese Medicine, Guangzhou, China; ^2^ Department of Orthopedics, Guangdong Provincial Second Hospital of Traditional Chinese Medicine, Guangzhou, China

**Keywords:** knee osteoarthritis, cellular senescence, publication trends, WoSCC, bibliometric

## Abstract

**Background:** Cellular senescence is associated with age-related pathological changes, senescent cells promote the development of knee osteoarthritis. A better understanding between knee osteoarthritis and cellular senescence may enhance the effectiveness of therapies that aim to slow or stop the progression of this disease.

**Purpose:** This study aimed to systematically analyze and visualize the publication trends, research frontiers and current research hotspots of knee osteoarthritis and cellular senescence by using bibliometrics.

**Methods:** The publication search was performed on the Web of Science Core Collection database for documents published from 1992 to 2023. VOSviewer, Citespace, R package Bibliometrix and Microsoft Office Excel were used to study the characteristics of the publications. The publication number, countries, institutions, authors, journals, citations and co-citations, keywords were analyzed.

**Results:** A total of 1,074 publications were analyzed, with an average annual growth rate of 29.89%. United States accounted for the biggest contributor, ranked first in publications and citations. Publications of this field were published in 420 journals, OSTEOARTHRITIS and CARTILAGE was the most influential. A total of 5,657 authors contributed to this research. The most productive author was Lotz, MK (n = 31, H-index = 22, Total citation = 2,619), followed by Loeser, R.F (n = 16, H-index = 14, Total citation = 2,825). However, the collaboration between authors was relatively weak. Out of the 1,556 institutions involved, 60% were from the United States. Scripps Research ranked first with 25 papers and a total of 2,538 citations. The hotspots of this field had focused on the pathomechanisms (e.g., expression, inflammation, apoptosis, autophagy, oxidative stress) and therapeutics (e.g., stem cell, platelet-rich plasma, transplantation, autologous chondrocytes, repair), and the exploration of Senolytics might be the important direction of future research.

**Conclusion:** Research on the cross field of knee osteoarthritis and cellular senescence is flourishing. Age-related pathomechanism maps of various cells in the joint and the targeted medicines for the senescent cells may be the future trends. This bibliometric study provides a comprehensive analysis of this cross field and new insights into future research.

## 1 Introduction

Knee osteoarthritis (KOA) is a chronic, degenerative disease commonly seen in clinic practice. Pathological changes involved the entire knee joint, including hyaluronic articular cartilage, subchondral bone, ligaments, joint capsule, synovium, sub-patellar fat pad, and periarticular muscles (Sharma, 2021). KOA progresses slowly and eventually leads to loss of mobility due to pain and impaired joint motion, especially in the elderly (Katz et al., 2021). KOA is not a disease driven by a single factor, but rather a heterogeneous syndrome. Risk factors such as joint injury, obesity, genetics, and metabolic bone disease are all associated with KOA (Yao et al., 2023), but the most common risk factor is age (Jeon et al., 2018). Numerous studies in recent years have shown that cellular senescence plays an important role in the development and progression of OA.

Cellular senescence was first proposed by Hayflick and Moorhead in 1961 (Hayflick and Moorhead, 1961). Cellular senescence is a stable terminal state in which the cell irreversibly leaves the cell cycle and enters a growth arrest (Gorgoulis et al., 2019). Telomere shortening, oxidative stress, and abnormal chromatin structure lead to nuclear DNA damage, resulting in cell cycle arrest and eventually cellular senescence (Roupakia et al., 2021). Cellular senescence is a stress response, mainly to remove damaged cells and eventually achieve tissue regeneration; however, with aging or continuous stimulation, the balance between senescence and regeneration is disrupted, and cellular senescence then becomes a problem rather than a normal procedure in tissue regeneration (Huang et al., 2022).

Numerous studies have found that cells in KOA exhibit a variety of aging-related phenotypes, like other organs, cells in the joint also show senescence and degeneration over time, and the number of senescent cells increases with aging (Diekman et al., 2018). However, the specific mechanism of cellular senescence related to KOA remains unclear, and many researchers have begun to explore targeting senescent cells as a treatment for KOA.

Bibliometrics analysis was first introduced by Prichard in 1969, has been widely utilized for assessing and quantifying publication data, including researchers. Countries and their cooperation, analysis of keywords, references, and co-citation can reveal the global trends and research hotspots. Outcomes from the bibliometric study can help researchers to identify the current research concerns to guide future research directions (Abramo et al., 2011).

However, bibliometric features have not been explored in the cross field of cellular senescence and KOA. The research on KOA and cellular senescence is progressing rapidly, it can be challenging for novice researchers to quickly grasp a thorough understanding of this field. Consequently, it is crucial to provide an overarching overview of research trends, focal areas, and significant contributions made by institutions and authors in this field. In this study, we aimed to analyze the publications in the cross field of KOA and cellular senescence from 1992 to 2023 through bibliometric methods. We conducted a thorough analysis to determine the current research status and knowledge base, aiming to assist researchers in understanding the historical hotspots and future research trends in this field.

## 2 Methods

### 2.1 Data collection and retrieval strategies

Web of Science Core Collection (WOSCC) is currently considered as one of the most comprehensive and authoritative databases for bibliometrics compared to databases such as Scopus and Pubmed (Zhu et al., 2020; Wu et al., 2021). We conducted a comprehensive search of all literature on KOA and cellular senescence using WOSCC on 10 May 2023. The search strategy was TS = Cellular Senescence OR Cell Aging AND TS = Knee Osteoarthritis, literature type = (Article OR Review), language = English. Upon completion of the search, the information data of the retrieved literature was selected as “Full Record and Cited References” and downloaded from the WOSCC database for further analysis. The literature information included authors’ name, publication year, institutions, countries, keywords, journals, citations and H-index, *etc.*

### 2.2 Data analysis and visualization

**FIGURE 1:**
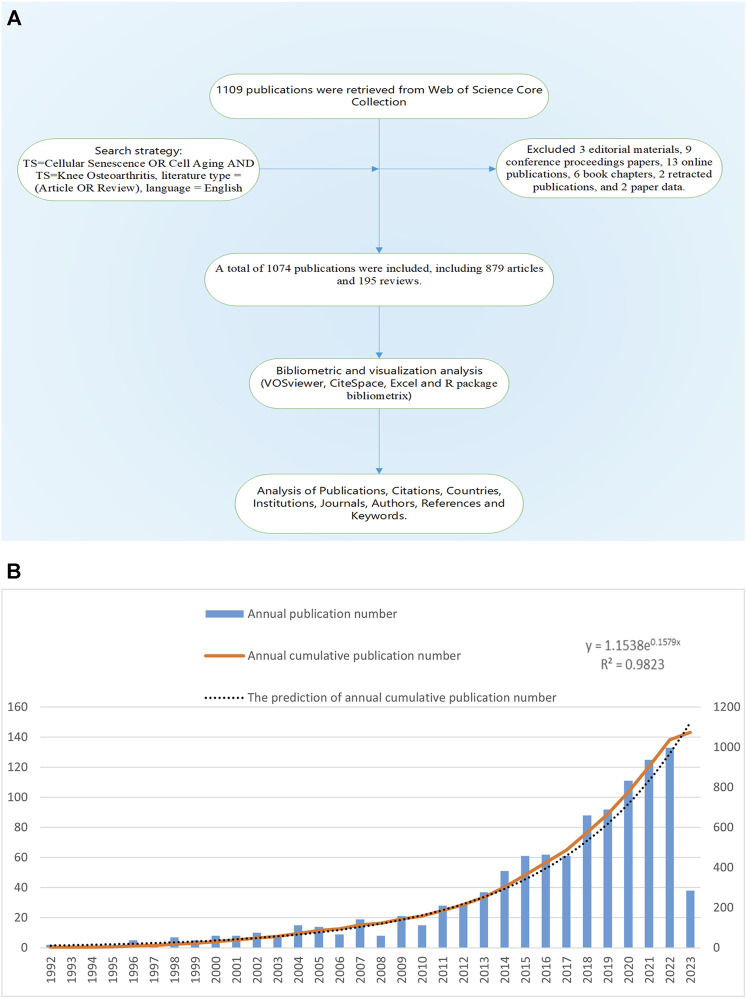
**(A)** Workflow diagram of this study. **(B)** The annual number of publications of KOA and cellular senescence from 1992 to 2023.

We used R package Bibliometrix, VOSviewer 1.6.18, Microsoft Office Excel 2021 and Citespace 5.8r5 for bibliometric analysis of the final included publications. R package Bibliometrix was used to extract basic information of all publications, such as the number of publications, types of publications, number of publications by authors, number of publications by journals, H-index of authors and journals, *etc.* VOSviewer was used for co-occurrence, co-authorship and co-citation analysis of keywords, countries, institutions and citations. The study utilized Microsoft Office Excel 2021 to analyze annual publication statistics and predict future trends. CiteSpace was used to detect the keywords and references with strong citation bursts, which were used as markers to identify research hotspots in different periods. Co-occurrence, co-authorship and co-citation analysis could identify high-frequency items and the links between them. Hotspots represented research directions that received significant attention from researchers. The citation burst analysis could identify references and keywords that experienced a notable increase in citations within a short period, indicated researchers’ interest during that time. Figure 1A showed the workflow diagram of this study.

## 3 Results

### 3.1 Publication summary

Based on the search criteria, 1,109 publications from 1992 to 2023 were retrieved, excluding 3 editorial materials, 9 conference proceedings papers, 13 online publications, 6 book chapters, 2 retracted publications, and 2 paper data. A total of 1,074 publications were included, written by 5,756 authors from 1,556 institutions and published in 420 journals, including 879 articles and 195 reviews.

The annual publication number of this field was low before 2001, rose slowly from 10 to 28 in 2002–2011, and entered a rapid growth period in 2011–2022, the annual publication number increased from 29 to 133, with an average annual growth rate of 29.89% (Figure 1B). The growth prediction model based on Microsoft Office Excel 2021:

$$y=1.1538e^{0.1579x},R^{2}=0.9823$$

The prediction model was highly consistent with the actual situation. It should be noted that 2023 was only 5 months away, so we did not include the publications of 2023. According to the prediction model, the number of publications in 2023 will reach 145, while the actual number of publications from January 1 to May 10 was 38, basically in line with the predicted amount.

### 3.2 Analysis of countries’ contribution

**FIGURE 2:**
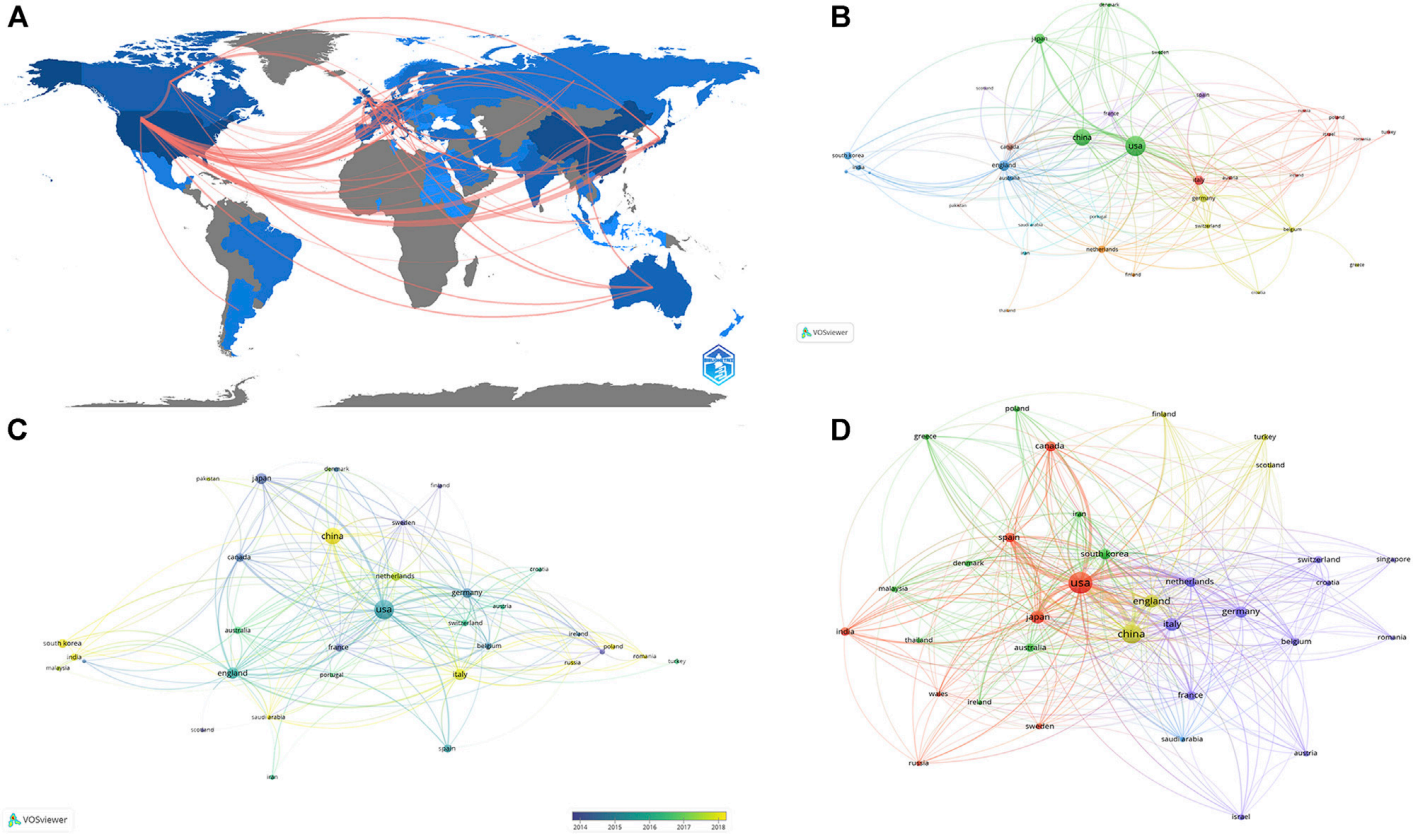
The analysis of countries **(A)** Shades of the color represented the publication number, the thickness of the line represents the frequency of cooperation. **(B, C)** The network map of countries, the size of the nodes represents the number of publications (B). The color represents the average appearing year (C). **(D)** The co-citation analysis of countries, the size of the nodes represents the number of total citations.

The publication number of different country/region was shown in Table 1, United States contributed the most (n = 265,24.7%), followed by China (n = 210,19.6%), the United Kingdom (n = 64, 6%), Japan (n = 62, 5.8%), and Italy (n = 59, 5.5%). Regarding the number of national citations, United States remained the leader in this area with 15,079 total citations and 56.90 average citations, China with 2,791 total citations and 13.30 average citations, the United Kingdom with 2,365 total citations and 37.00 average citations, and Japan with 20,19 total citations and 32.60 average citations. It was worth noting that the publication number of Netherlands was only 32, but with 2,473 total citations and 77.30 average citations, ranking first among all countries. As shown in Figure 2A, United States cooperated the most with other countries, including China, Japan, Germany, the United Kingdom, and the Netherlands. Table 2 showed the information of the top ten countries with most cooperation frequency.

**TABLE 1:** The top ten cooperation frequency of countries.

From	To	Frequency
United States	CHINA	27
United States	JAPAN	24
United States	GERMANY	15
United States	UNITED KINGDOM	14
United States	NETHERLANDS	13
ITALY	UNITED KINGDOM	12
United States	CANADA	12
United States	ITALY	10
United States	SPAIN	9
United Kingdom	CANADA	8

**TABLE 2:** General information of top ten countries with most publications.

Country	Publications	Total citations	Average citations
United States	265	15,079	56.90
CHINA	210	2,791	13.30
UNITED KINGDOM	64	2,365	37.00
JAPAN	62	2019	32.60
ITALY	59	1,596	27.10
KOREA	48	1,488	31.00
GERMANY	37	1,237	33.40
NETHERLANDS	32	2,473	77.30
CANADA	28	675	24.10
SPAIN	25	952	38.30

### 3.3 Analysis of higher-impact journals

**FIGURE 3:**
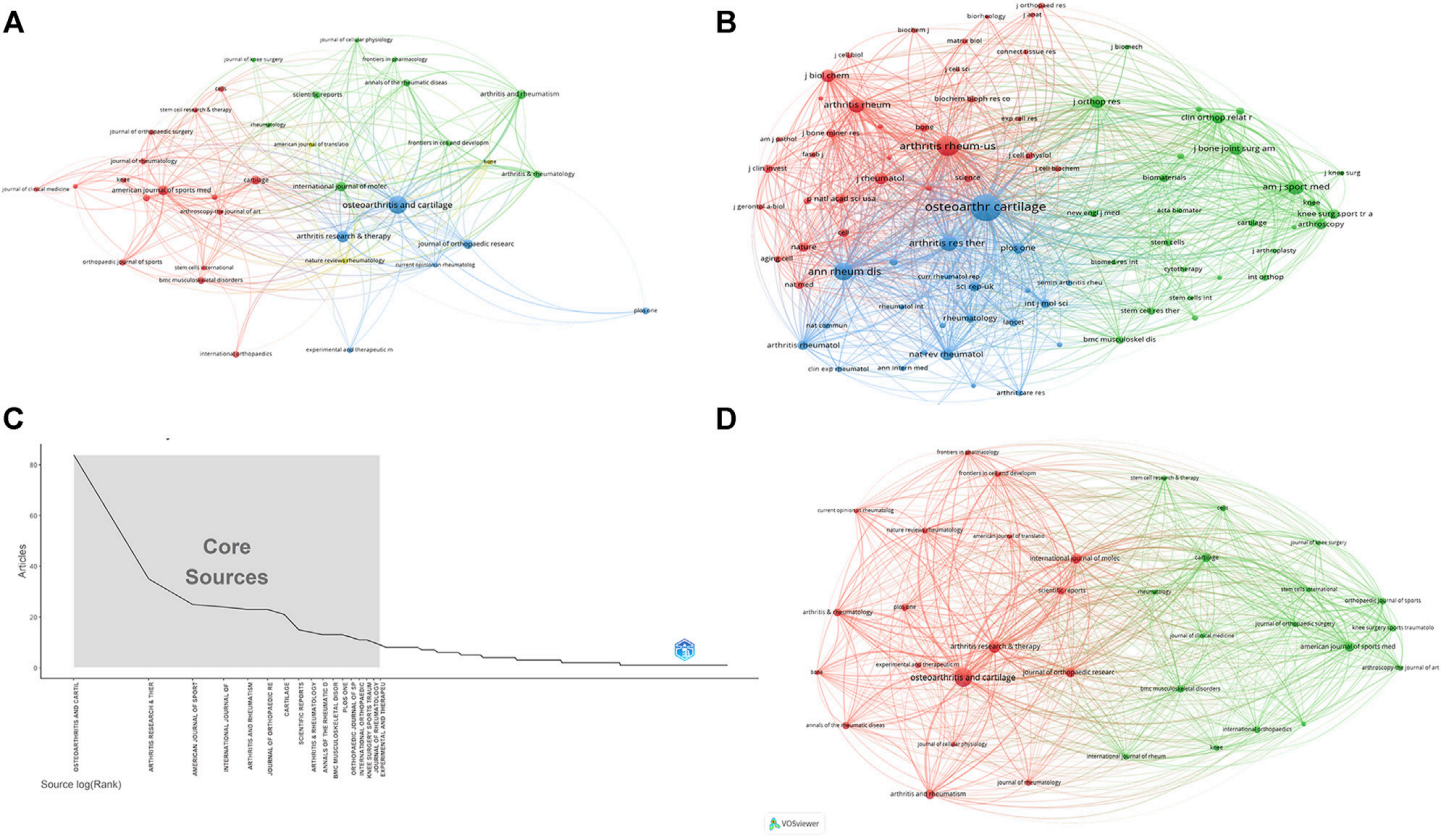
The analysis of journals **(A)** The analysis of publications, the size of the nodes represents the publication number of journals. **(B)** The analysis of citations, the size of the nodes represents the total citations of journals. **(C)** Core journals according to the Bradford’s Law. **(D)** The co-citation analysis of journals, the size of the nodes represents the total citations of journals.

Research of KOA and cellular senescence was published in 420 journals. According to Bradford’s theorem (Figure 3C), the 17 core journals were those with 9 or more publications. Table 3 summarized the basic information of the top ten journals with high publication number. Based on publication number and H-index of journals, OSTEOARTHRITIS and CARTILAGE was the most influential, with 84 publications (H-index = 37, Total citations = 5,095, UK), including 76 articles and 8 reviews, indicated that the journal always paid attention to high-quality research and had been widely recognized in the cross field of KOA and cellular senescence.

**TABLE 3:** The general information of top ten most productive journals.

Sources	Articles	H-index	Total citations	If
OSTEOARTHRITIS AND CARTILAGE	84	37	5,095	7.507
ARTHRITIS RESEARCH and THERAPY	35	21	1700	5.606
AMERICAN JOURNAL OF SPORTS MEDICINE	25	17	2,283	7.1010
INTERNATIONAL JOURNAL OF MOLECULAR SCIENCES	24	8	411	6.208
ARTHRITIS AND RHEUMATISM	23	21	2,202	5.431
JOURNAL OF ORTHOPAEDIC RESEARCH	23	16	866	3.102
CARTILAGE	21	8	308	3.117
SCIENTIFIC REPORTS	15	8	449	40,996
ARTHRITIS and RHEUMATOLOGY	14	12	692	15.483
ANNALS OF THE RHEUMATIC DISEASES	13	11	987	27.973

### 3.4 Analysis of institutions

**FIGURE 4:**
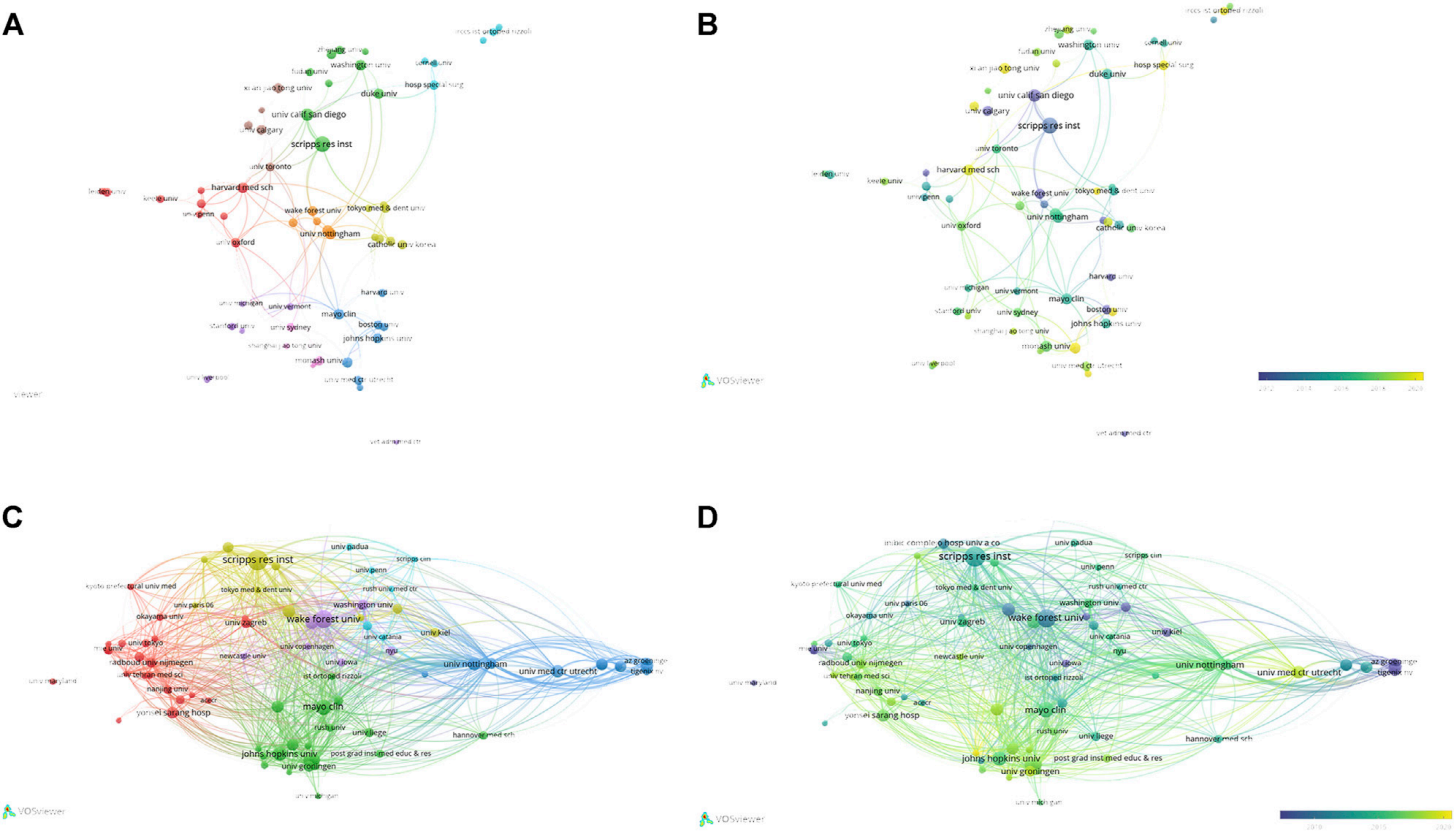
The analysis of institutions **(A, B)** The network map of institutions, the size of the nodes represents the number of publications. The color of the nodes represents the average appearing year (B). **(C, D)** The analysis of institutions’ citations, the size of the nodes represents the number of citations. The color represents the average appearing year (D).

A total of 1,556 institutions published 1,074 publications. Table 4 showed the top ten institutions with highest publication number. Among these institutions, 60% were from United States, and the others were two from China, one from the United Kingdom, and one from Australia. Scripps Research ranked first with 25 papers and 2,538 total citations, followed by the University of California, San Diego (n = 17,TC = 1,008), and the University of Nottingham (n = 13, Total citations = 1,046), the University of Washington (n = 13,TC = 649), and Xi ‘an Jiaotong University (n = 13,TC = 268). Table 5 showed the difference in high publications institutions and high citations institutions, High citation institutions generally started their research earlier, such as Wake Forest University, Johns Hopkins University, Scripps Research, Mayo Clinic, Cornell University, *etc.* Figure 4A showed a strong cooperative relationship between Scripps Research, University of California, San Diego, and University of Washington, which had a higher number of citations and publications.

**TABLE 4:** The top ten institutions with most publications.

Country	Institution	Publication	Total citations
United States	Scripps Research	25	2,538
United States	University of California, San Diego	17	1,008
United Kingdom	University Of Nottingham	13	1,046
United States	University of Washington	13	649
CHINA	Xi ‘an Jiaotong University	13	268
United States	Harvard Medical School	12	154
United States	Mayo Clinic	12	1,486
AUS	Monash University	12	484
United States	RUSH University	12	435
CHINA	Southern Medical University	12	153

**TABLE 5:** The top ten institutions with most citations.

Country	Institution	Publications	Citations
United States	Scripps Research	25	2,538
United States	Wake Forest University	10	1989
United States	Mayo Clinic	12	1,486
United States	Johns Hopkins University	11	1,238
United States	Cornell university	7	1,101
United Kingdom	University Of Nottingham	13	1,046
Netherlands	Utrecht University	10	1,018
United States	University of California, San Diego	17	1,008
United States	North Carolina State University	8	932
KOREA	Yonsei Sarang Hospital	10	874

### 3.5 Analysis of authors

**FIGURE 5:**
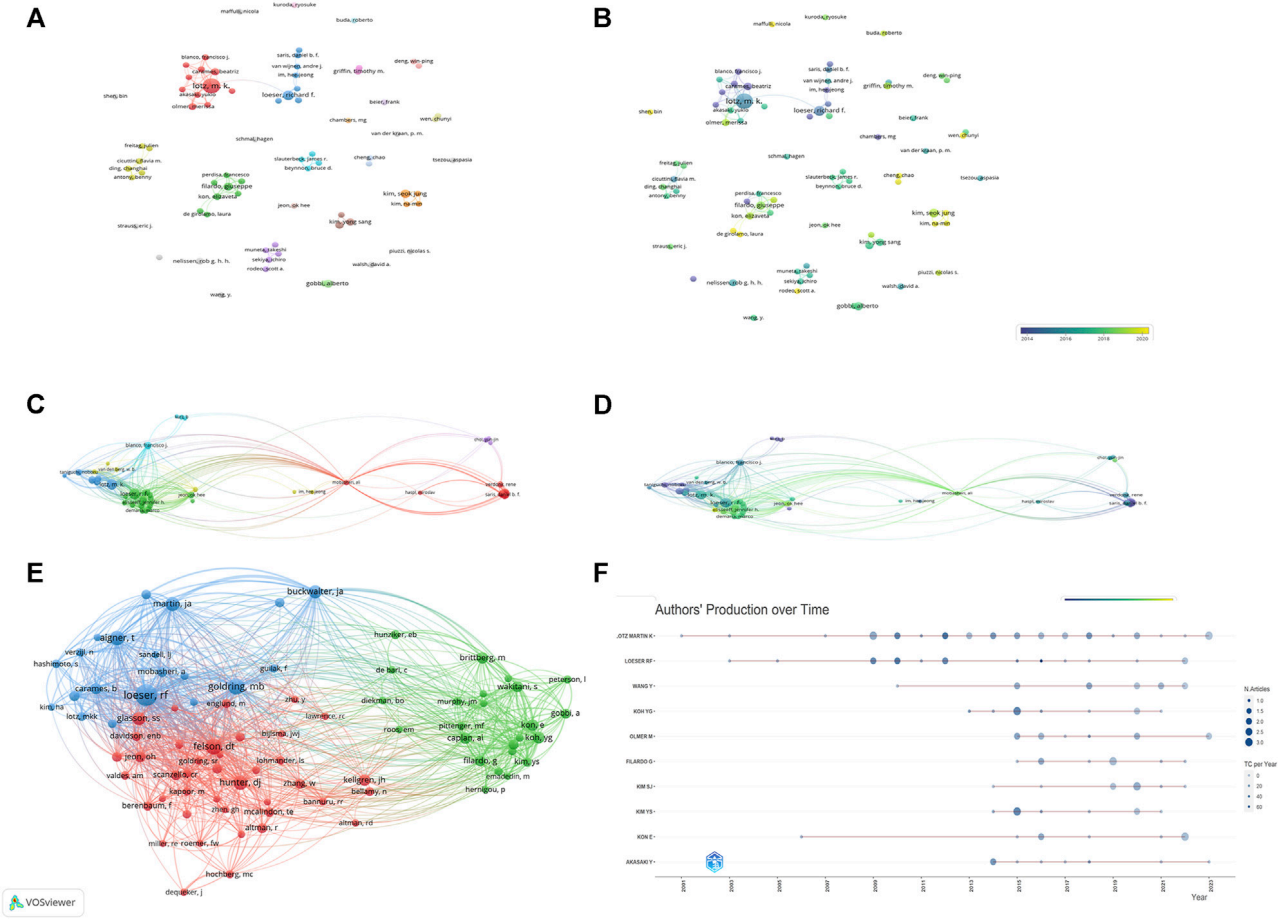
The analysis of authors **(A, B)** The network map of authors, the size of the nodes represents the number of publications. The color of the nodes represents the average appearing year (B). **(C, D)** The citation number of authors, the size of the nodes represents the number of citations. The color represents the average appearing year (D). **(E)** The bibliographic coupling of authors, the size of the nodes represents the number of publications. **(F)** The publication over time of top ten productive authors.

5,676 authors had published 1,074 literature in this field. Table 6 showed the information of top ten authors with the highest publication number. Lotz M.K was the leader in this field based on publication number, citations, and H-index, and had long been deeply involved in the research of KOA and cellular senescence. Figure 5A showed the analysis of authors, with some collaboration only between highly influential authors Lotz M.K, Loeser R.F, Carames Blanco F.J. The collaboration between other authors was weak, indicated that researchers in this field need to cooperate more closely and frequently to promote the development of this field.

**TABLE 6:** The top ten authors with most publications.

Author	Publications	Total citations	H-index
LOTZ M K	31	2,619	22
LOESER RF	16	2,825	14
OLMER M	10	445	8
KOH YG	10	874	8
FILARDO G	9	230	7
KON E	9	323	7
CARAMES B	8	1,276	7
AKASAKI Y	8	472	7
GOBBI A	8	461	7
WALSH DA	7	419	7

### 3.6 Analysis of citations and co-citations

**FIGURE 6:**
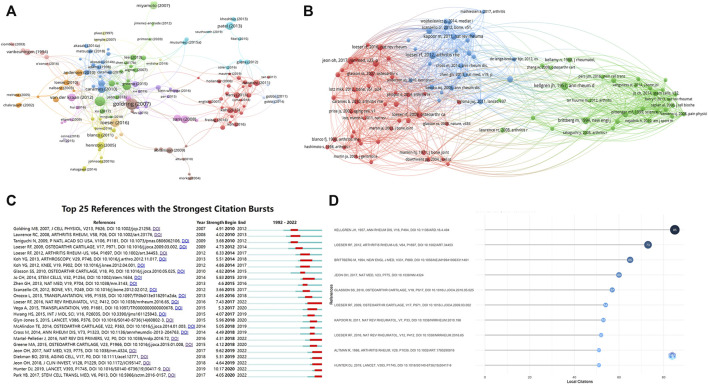
The analysis of citation and Co-citation **(A)** Network map of total citations of publications. **(B)** Network map of co-cited references. **(C)** Top 25 references with strongest citation bursts. **(D)** The top ten references with most local citations.

A total of 205 publications with more than 50 citations were identified. Table 7 showed the top ten publications with highest citations. Figure 6A showed the network map of total citations of publications. Figure 6B showed the network map of co-cited references. Additionally, a co-citation analysis of 39,069 references was conducted, and 99 literatures were cited more than 20 times. The first was published by Kellgren J.H and Lawrence J.S in 1957, proposed the Kellgren-Lawrence grading scoring system of osteoarthritis, which classified KOA from mild to severe as grade 0, I, II, III, and IV based on the features of x-ray (Kellgren and Lawrence, 1957). The second was published by Loeser R.F et al., in 2012, which outlined the pathological changes of joint tissues in KOA and the mechanisms of these changes (Loeser et al., 2012). The third was published by Brittberg M et al., in 1994, the author used cultured autologous chondrocytes for the repair of deep cartilage defects on the tibiofemoral articular, achieved good results (Brittberg et al., 1994). The fourth was published by Jeoh et al., in 2017, which found that selective removal of local senescent cells can significantly promote the regeneration of patient cartilage (Jeon et al., 2017). The fifth was published by Glasson S.S et al., in 2010, which proposed the OARSI score of mouse osteoarthritis tissue (Glasson et al., 2010). We also conducted the top 25 references with citation burst, to identify Figure 6D showed the top ten references with most local citations the references with a remarkable citation in a relatively short period of time (Figure 6C).

**TABLE 7:** The top ten most cited publications.

Title	Author	Journal	Year	Total citations
Osteoarthritis	GOLDRING MB	Cellular Physiology	2007	953
Local Clearance of Senescent Cells Attenuates The Development of Post-traumatic Osteoarthritis and Creates a Pro-Regenerative Environment	JEON OH	NATURE MEDICINE	2017	721
Ageing and the Pathogenesis of Osteoarthritis	LOESER RF	Nature Reviews Rheumatology	2016	526
Characterized Chondrocyte Implantation Results in Better Structural Repair when Treating Symptomatic Cartilage Defects of the Knee in a Randomized Controlled Trial *versus* Microfracture	SARIS DBF	American Journal of Sports Medicine	2008	455
Autophagy Is a Protective Mechanism in Normal Cartilage and Its Aging-Related Loss Is Linked With Cell Death and Osteoarthritis	CARAMES B	ARTHRITIS and RHEUMATISM	2010	453
Chondrocyte Hypertrophy And Osteoarthritis: Role in Initiation And Progression Of Cartilage Degeneration?	VAN DER KRAAN PM	OSTEOARTHRITIS AND CARTILAGE	2012	450
Treatment With Platelet-Rich Plasma Is More Effective Than Placebo for Knee Osteoarthritis: A Prospective, Double-Blind, Randomized Trial	PATEL S	American Journal of Sports Medicine	2013	447
Aging And Osteoarthritis: The Role of Chondrocyte Senescence and Aging Changes In The Cartilage Matrix	LOESER RF	OSTEOARTHRITIS AND CARTILAGE	2009	426
Oxygen and reactive oxygen species in cartilage degradation: friends or foes?	HENROTIN Y	OSTEOARTHRITIS AND CARTILAGE	2005	370
Treatment of Symptomatic Cartilage Defects of the Knee: Characterized Chondrocyte Implantation Results in Better Clinical Outcome at 36 Months in a Randomized Trial Compared to Microfracture	SARIS DBF	American Journal of Sports Medicine	2009	290

### 3.7 Analysis of keywords

**FIGURE 7:**
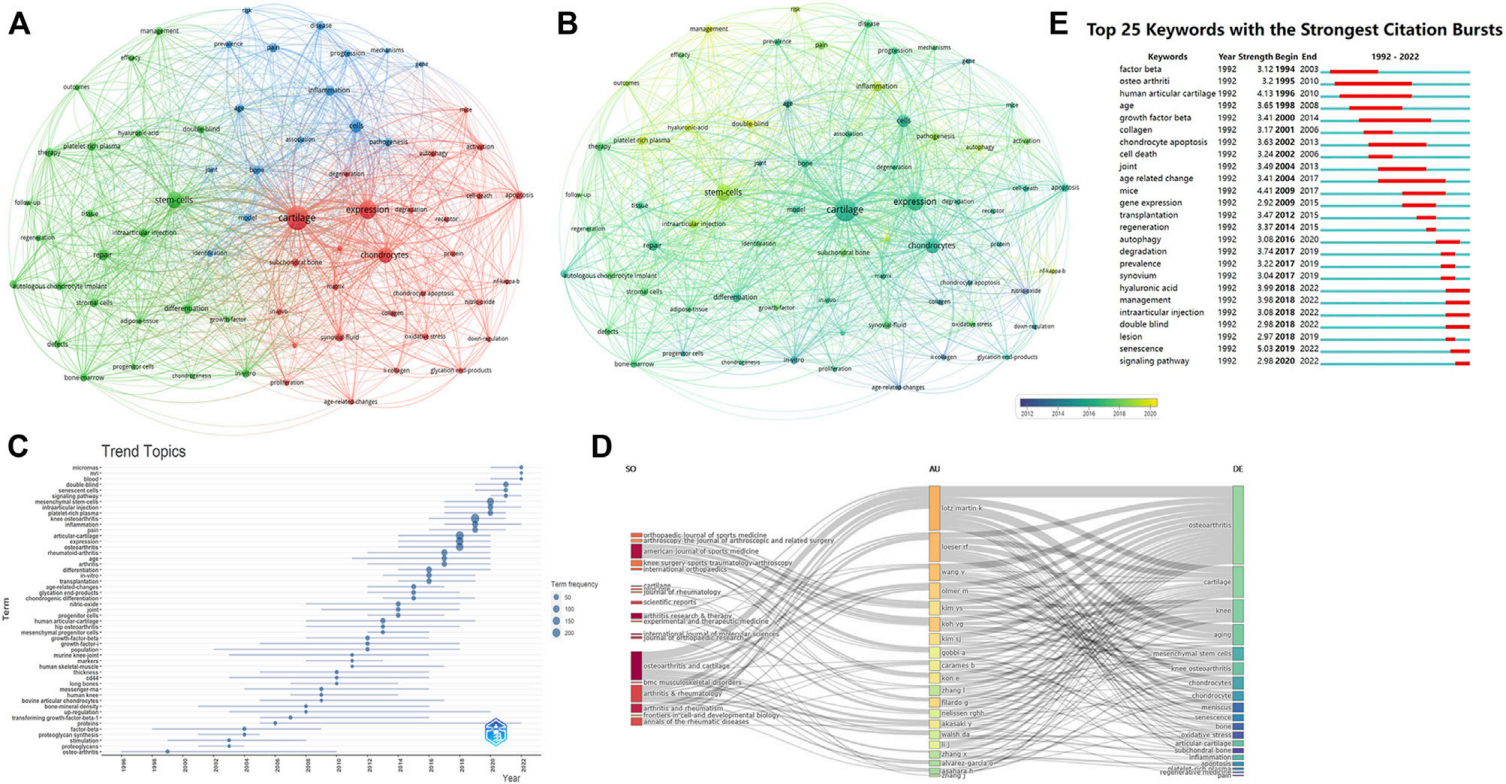
The analysis of keywords **(A, B)** Network map of keywords, the size of the nodes represents the frequency. The color represents the average appearing year (B). **(C)** The trend topics based on keywords generated by R package Bibliometric. **(D)** The three-plot field of journals, authors and keywords. **(E)** Top 25 keywords with strongest citation bursts.

Figure 7A illustrated the network analysis of keywords, the node size represented the occurrence frequency, the distance of two nodes reflected their association strength, and the nodes with a closer distance were classified as the same cluster. Knee osteoarthritis and cell senescence which appeared most frequently in this study were not shown. Red represented cluster I, which showed the tissues, cells, and metabolic processes associated with cell senescence. Such as cartilage, subchondral bone, chondrocytes, anterior cruciate ligament, synovial fluid, collagen, and matrix, *etc.* Blue represented cluster II, the main nodes were inflammation, progression, mechanism, etiology, epidemic, risk. Green represented cluster III, mainly focused on clinical treatments, such as stem cells, intra-articular injections, autologous chondrocyte transplantation, repair, and platelet-rich plasma.

In overly visualization the color from blue to yellow represented the early and late appearance of the keywords. The research on cartilage, chondrocytes, collagen, and nitric oxide appeared earlier, and researchers had recently targeted autophagy, inflammation, stem cells. Figure 7C showed the topic trends, and the results were generally consistent with the overly visualization. The top 25 keywords with the strongest citation burst were also conducted, in order to show the historical research trend of this field (Figure 7E).

In addition, we also constructed a three-field plot of journals, authors and keywords (Figure 7D), and the results were in line with the above analysis. Lotz M.k and Loeser R.F were most closely related to this cross field, and their research was mainly focused on cartilage changes. The journals most closely associated with this field were Osteoarthritis and Cartilage, American Journal of Sports Medicine, and Arthritis and Rheumatism.

### 3.8 Analysis of research topic and types in different countries

**FIGURE 8:**
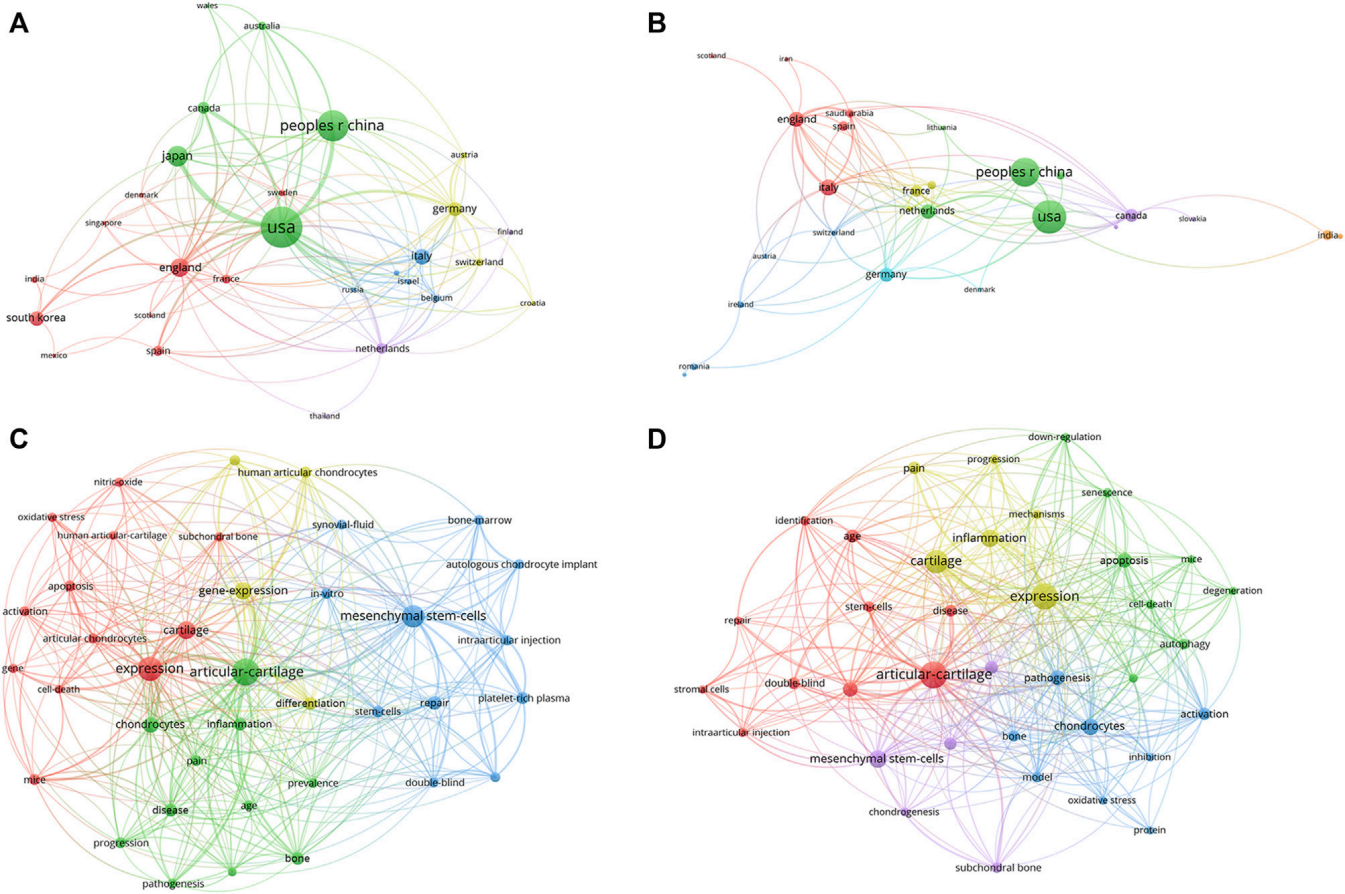
The analysis of research topic and types in different countries **(A)** Countries’ network map of articles, the size of the nodes represents the frequency. **(B)** Countries’ network map of reviews, the size of the nodes represents the frequency. **(C)** The keywords network map of United States, the size of the nodes represents the frequency. **(D)** The keywords network map of China, the size of the nodes represents the frequency.

In order to show the research topics and types among countries, we separately extracted the bibliometric information of articles, reviews and countries with a large number of publications. Due to the publication number of countries other than the United States and China were relatively small, we conducted keywords network analysis of the United States and China. Figure 8 illustrated the co-authorship analysis of articles and reviews among countries, and keywords network analysis for United States and China. Our findings showed that the United States contributed the largest number of articles, while the review number of China was in the same level with the United States. Indicated that China should focus on improving the quality and quantity of studies. Keywords network analysis indicated that the United States had conducted more extensive research in this field, with deep cultivation in pathomechanisms and therapeutics, while the research topics of China were mainly focused on pathomechanisms.

## 4 Discussion

### 4.1 Summary of findings

Bibliometric analysis was a method of evaluating importance and influence of publications from various aspects, providing a reliable analysis of bibliometric parameter for researchers in a certain field (Ellegaard and Wallin, 2015). The analysis of the annual publication number represented the popularity of research in this field (Wen et al., 2022). The annual publication number in this field began to grow rapidly after 2010 Until the date of our bibliometric data was downloaded, the number of publications in 2023 was basically in line with the prediction model, indicated that this field had been paid more and more attention from researchers.

With the continuous progress of medical technology and social productivity, many countries had entered the stage of aging population, and the incidence of KOA increased year by year, which brought heavy economic burden on both society and patients (Giorgino et al., 2023). The top ten countries in terms of publication number and citations were all developed countries except China, which might be related to the investment of scientific research funding and the increase of incidence in these countries. As the biggest developing country and the second largest population in the world, China had a large number of KOA patients. The number of publications in China might be related to these aspects. Although the number of publications in China had been growth in recent years, the citations was relatively low, suggested that Chinese researchers might need to focus on improving the quality of their research to enhance their impact and contribution in this field.

The country with the largest contribution in this cross field was United States, which was far ahead of other countries, and had conducted more collaborations with other countries. This might be related to the fact that United States had the highest healthcare spending globally. It was worth noting that the number of publications of Netherland was not prominent, but the average citations of Netherland ranked first, indicated that the quality of these research was relatively high.

Among the top ten institutions, six were from the United States, two were from China, and the rest were from the United Kingdom and Australia. The institution with the highest publications number was Scripps Research (n = 25, Total citation = 2,538), which was the absolute leader in this field. It was worth noting that Wake Forest University, with only ten publications, but the citation number had reached 1989. The network map of institutions showed that the institutions from the United States and Europe had conducted a longer period of time in this cross field. In recent years, some institutions in China had also made a lot of exploration in this field, such as Xi ‘an Jiaotong University and Southern Medical University. Although the publication number of these institutions was high, but the citation number was quite low. On the one hand, it might be due to the short time since publication, which had not form a large number of citations. On the other hand, it might be due to the low quality of publications, which had not form a huge impact in this filed.

The publication number of Scripps Research might be related to the core author in this field, Lotz M.K (n = 31, H-index = 22, Total citation = 2,619), who mainly focused on joint senescence, the pathogenesis of osteoarthritis and drug discovery. The second highest contributor in this field was Loeser R.F (n = 16, H-index = 14, Total citation = 2,825). It was worth noting that although the publication number of Loeser R.F was much lower than Lotz M.K, there were 2,825 citations of Loeser’s publications, ranking first among all authors. The two reviews in 2009 and 2016 were cited for 426 times and 526 times, respectively (Loeser, 2009; Loeser et al., 2016). Loeser mainly focused on the mechanisms related to joint tissue destruction in osteoarthritis. The analysis of co-authorship showed that the correlation between authors was low. Indicated that it was necessary to establish stronger cooperation between researchers in this field, which might be crucial to the development of this field.

The publications of KOA and cellular senescence were published in 420 different journals. According to Bradford Theorem, 18 core journals accounted for 4.28% of all journals, with a total of 356 publications accounting for 33% of the total publications. The most influential was OSTEOARTHRITIS and CARTILAGE (n = 84, H-index = 37, Total citation = 5,095), ranking first among all the journals. Among the other journals, ARTHRITIS and RHEUMATISM and AMERICAN JOURNAL of SPORTS MEDICINE had high impact, with large number of publications and citations. Among the top ten journals in terms of publication number, only ARTHRITIS and RHEUMATOLOGY had an impact factor higher than 10. Indicated that it was challenging to publish literature of this field on high-impact factor journals.

### 4.2 Hot topic in basic and clinical research

Since Hayflick and Moorhead proposed the concept of cellular senescence in 1961, many researchers had investigated the pleiotropic role of cellular senescence as a driver of diseases, including tumors, atherosclerosis, cardiac dysfunction, and renal insufficiency (Wang and Bennett, 2012; Docherty et al., 2019; Chen et al., 2022b; Schmitt et al., 2022). As one of the hallmarks of aging, the increased burden of senescent cells in various tissues was the main pathogenic factors of aging-related diseases (Tominaga, 2015). The current understanding of cellular senescence in KOA was that the senescence of all tissues would impact on the degenerative changes of the whole joint (Wu et al., 2022). The network analysis of keywords revealed that the most frequent keywords were cartilage, expression, stem cells, and chondrocytes. And all the keywords were divided into three clusters, in which red was cluster I, green was cluster II, and blue was cluster III.

The combination of cluster I and cluster II were roughly the internal structure of the joint and the pathological changes in the development of KOA. Such as cartilage, chondrocytes, bone, subchondral bone, joint synovial fluid, anterior cruciate ligament, expression, inflammation, oxidative stress, autophagy, apoptosis, mechanism, pathology, *etc.* Initially, researchers considered KOA as a matter of mechanical damage, in contrast, KOA was caused by an imbalance of the whole joint tissues. The focus of researchers was on the senescent associated secretory phenotype (SASP) of senescent cells; the effect of senescent cells on surrounding cells; the upstream regulatory mechanisms of cellular senescence. In this process, the pathological changes of articular cartilage associated with aging were most concerned by researchers, because the most significant pathological changes in KOA were degeneration of chondrocytes and degradation of the extracellular matrix. Articular chondrocytes were usually in a state of low proliferation, after joint injury, chondrocytes could enhance their proliferation capacity to repair cartilage, but it would also make chondrocytes more prone to cell senescence, thereby promoting the development of KOA (Ashraf et al., 2016; Loeser et al., 2016; He and Sharpless, 2017).

However, some researchers believed that the effect of chondrocyte senescence on KOA might be driven by SASP factors, rather than the decline of chondrocytes proliferation (Greene and Loeser, 2015; Chen et al., 2022a). SASP referred to the secretion of specific bioactive molecules by senescent cells that could induce a range of physiological responses in the surrounding microenvironment, including inflammation, cell cycle arrest, and tumors (Birch and Gil, 2020). Several common SASP factors such as MCP1, IL1, IL6, MMP3, and MMP13, which contributed to the inflammatory microenvironment of KOA (Liu et al., 2021). Chronic inflammation might lead to the senescence of other cells in the joint, extracellular matrix breakdown, synovial inflammation and subchondral bone remodeling (Cai et al., 2020). Moreover, some inflammatory cytokines in synovial fluid might also be the source of pain in KOA (Nees et al., 2019).

Xu and others found that the transplantation of senescent fibroblasts into the knee joint of mice leads to cartilage degeneration, osteophytes formation and affect the mobility of mice, indicated that the knee joint microenvironment was altered by senescent cells through paracrine effects and induce pathological changes (Xu et al., 2017). Therefore, many researchers believed that a better understanding of the pathogenesis of KOA and the expression of SASP factors in the joint tissue might be a promising research direction.

Oxidative stress was also a hot topic in this field, as mitochondrial dysfunction in senescent cells led to an increase in reactive oxygen species (ROS), which in turn caused oxidative stress (Erusalimsky, 2020; Ebert et al., 2022). Oxidative stress was thought to be a driver of catabolic and anabolic imbalance in cartilage, leading to degradation of cartilage extracellular matrix and causing apoptosis (Bolduc et al., 2019; Kulkarni et al., 2021). Some researchers had found that the progression of KOA can be delayed by inhibiting oxidative stress (Feng et al., 2019; Wang et al., 2020). Indicated that inhibiting oxidative stress was also a potential strategy for the treatment of KOA.

Autophagy protected the body from cellular senescence by removing damaged organelles and long-lived macromolecules (Mizushima and Levine, 2020; Gong et al., 2023). Autophagy was an indispensable mechanism to maintain the stability of the intracellular environment. In articular cartilage, the role of autophagy was particularly important in maintaining chondrocyte homeostasis and function due to the low proliferation rate of chondrocytes (Kao et al., 2022). Carames and Martin Lotz found that autophagy was a self-protective mechanism of cartilage and that impaired autophagy with cellular senescence led to chondrocyte apoptosis (Carames et al., 2010). And they found that rapamycin could reduce the severity of osteoarthritis by activating autophagy, indicated that activating autophagy in chondrocytes through drugs might be an effective treatment for KOA (Lopez de Figueroa et al., 2015). In 2023, Irene Lorenzo-Gómez and others found a key chaperone for chondrocyte autophagy, HSP90A, defective chaperone mediated autophagy might be crucial to KOA (Lorenzo-Gomez et al., 2023).

In the pathological process of KOA, senescent chondrocytes might affect the chondrogenic differentiation potential of bone marrow derived Mesenchymal stem cells (MSCS), and senescent MSCs might lose their immunomodulatory effect, thereby promoting the development of KOA (Cao et al., 2019; Malaise et al., 2019). Mateos J and Arufe MC revealed that the chondrogenic differentiation of human MSCs was negatively affected by Lamin A deregulation, which in turn disrupted the oxidative stress balance in MSCs (Mateos et al., 2013). Murphy and others used Luciferase to label stem cells in different types of mouse articular cartilage and found that the number of stem cells decreased significantly with age (Murphy et al., 2020).

The cluster III mainly focused on therapeutic modalities, such as stem cells, repair, platelet-rich plasma, intra-articular injection, and transplantation. Many researchers had revealed the important pathological role of stem cell senescence in KOA and also provided a theoretical basis for endogenous stem cell therapy for KOA (Prajwal et al., 2022). In recent years, more and more researchers believed that exosomes secreted by MSCs also play a role in the treatment of osteoarthritis (Yu et al., 2022). Miriam Morente-López and others found that the extracellular vesicles treatment of MSCs was more effective than miR-21−MSCs themselves in reducing systemic inflammation in KOA (Morente-Lopez et al., 2022). Researchers found that local intra-articular injection of MSCs can promote the regeneration and repair of cartilage tissue and reduce the degeneration caused by KOA (Tong et al., 2020). MSCs were able to regulate local inflammation, apoptosis and proliferation of cells and promote the repair of bone and cartilage by secreting exosomes, growth factors, cytokines, anti-inflammatory factors and other bioactive molecules (Zhang et al., 2021). There are currently 140 clinical studies of stem cell therapy for KOA registered on clinicaltrial.gov (Clinicaltrials, 2023).

Platelet-rich plasma (PRP) was a platelet concentrate extracted from autologous blood by centrifugation, intra-articular injection of PRP could induce cell migration and proliferation, promote the formation of cell matrix and cartilage regeneration, inhibit chondrocyte apoptosis, ameliorate inflammatory microenvironment (Andia et al., 2021; Szwedowski et al., 2021). Although many researchers had found the positive effect of PRP for the treatment of KOA, some researchers found that there were some heterogeneities in the clinical efficacy of PRP, it might be related to the preparation of PRP, concentration of each component and frequency of treatment, rest time, etc (Harrison, 2018). Therefore, it was of great significance to formulate an international standard for the preparation and application of PRP (Anitua and Prado, 2019).

Some researchers had also explored cartilage and chondrocyte transplantation. However, there were some problems in clinical application, such as the difficulty of obtaining autologous cartilage and the rejection of allogeneic cartilage (Lindahl, 2015; Sato et al., 2019).

### 4.3 The future research trends

To further explore the research dynamics in the cross field of KOA and cellular senescence, we conducted the analysis of keywords and citations bursts. After comprehensive analysis, we found that a publication by Joen H and others in 2017, had received widespread attention (Jeon et al., 2017). This publication had been cited multiple times in a relatively short period of time, with 60 local citations and 721 global citations. They found that intra-articular injection of the Senolytic UBX0101 could selectively removes local senescent cells, thereby alleviating post-traumatic osteoarthritis and creating an environment for cartilage regeneration. Since then, many researchers had explored the therapeutic effect of other Senolytics. In 2019 Garrett A and others found that navitoclax (ABT-263), a dual BCL-2 and BCL-XL inhibitor, could eliminate senescent chondrocytes expressing high levels of p16 by apoptosis (Sessions et al., 2019). George Batshon et al. found that combined application of Navitoclax and UBX0101 could reduce the Serum NT/CT SIRT1 ratio in ACLT mice by eliminating senescent cells, thus alleviating KOA (Batshon et al., 2020). High-throughput drug screening could be used to discover new Senolytics, unveiling new mechanisms that contribute to the treatment of KOA. Nogueira-Recalde U et al. screened over 1,000 compounds for Senolytic therapies in human chondrocytes, found that Fenofibrate, an agonist of flavonoids and peroxisome proliferator-activated receptor-α (PPARα), was able to induce apoptosis in SA-β-gal-positive chondrocytes (Nogueira-Recalde et al., 2019). This discovery led the authors to investigate PPARα expression in KOA, and they found that the expression of PPARα was reduced in the blood and knee cartilage of patients with OA. Flavonoids that could activate sirtuins, such as Fisetin, were linked to longevity and inhibit IL-1β induced inflammation in osteoarthritic chondrocytes. Fisetin was evaluated in clinical trials for efficacy in alleviating osteoarthritis symptoms by reducing senescence burden in cartilage.

Although pharmacological approaches to treat cellular senescence related KOA seemed promising, potential side effects and differences in drug efficacy remained concerns (Bai et al., 2022). Even if senescent cells were responsible for KOA progression, prematurely eliminating these cells or preventing paracrine signaling might impede the initial healing of tissues (Yun et al., 2015). Several studies had shown that Senolytics can target to eliminate senescent chondrocytes, the subsequent maintenance of cartilage homeostasis was still unknown (Dai et al., 2020; Miura et al., 2022). Although Senolytic had achieved positive effects in the laboratory, there was still a certain distance from clinical application. Therefore, the construction of aging mechanism maps of various tissues and cells such as cartilage, synovium, subchondral bone and stem cells might be of great significance to reveal the mechanism of KOA and find new therapeutic targets.

## 5 Limitations

There were some limitations to our study. Firstly, the publications were only retrieved from one database, no search was conducted in other databases such as Pubmed, Embase, Cochrane library, so there might be a certain degree of omission in the data. Secondly, we only collected English publications, which might cause selection bias, leading to certain errors in the results. Finally, the cross field of cellular senescence and KOA was constantly evolving and new publications were being published every day, some high-quality studies published in recent years might have been overlooked due to limited citation.

## 6 Conclusion

In conclusion, this study is the first bibliometric analysis of the intersection of KOA and cellular senescence, we summarize the current research status, countries/regions, authors, keywords, and hotspots of this field. A strong ascending trend for the publication number is observed, and it may be a hint that researchers’ interest and attention in this field are increasing. Senolytics may be the up-to-date research frontiers. Our analysis provides a scientific perspective on the cross filed of KOA and cellular senescence for relevant researchers, funding agencies, and policymakers.

## Data Availability

The raw data supporting the conclusions of this article will be made available by the authors, without undue reservation.
